# Predictive value of a nomogram for melanomas with brain metastases at initial diagnosis

**DOI:** 10.1002/cam4.2644

**Published:** 2019-10-27

**Authors:** Hong Liu, Yan‐Bo Xu, Cheng‐Cheng Guo, Ming‐Xin Li, Jia‐Li Ji, Rong‐Rong Dong, Ling‐Ling Zhang, Xue‐Xin He

**Affiliations:** ^1^ Department of Medical Oncology The Second Affiliated Hospital of Zhejiang University Hangzhou Zhejiang China; ^2^ Department of Surgical Oncology The Second Affiliated Hospital of Zhejiang University Hangzhou Zhejiang China; ^3^ State Key Laboratory of Oncology in South China Department of Neurosurgical Oncology Sun Yat‐Sen University Cancer Center Guangzhou Guangdong China; ^4^ College of Medicine Upstate Medical University New York NY USA; ^5^ Department of Oncology Affiliated Cancer Hospital of Nantong University Nantong Jiangsu China; ^6^ Department of Internal Medicine The Children's Hospital of Zhejiang University Hangzhou Zhejiang China; ^7^ Department of Oncology International Hospital of Peking University Beijing China; ^8^ Division of Internal Medicine The University of Texas MD Anderson Cancer Center Houston TX USA

**Keywords:** melanoma, melanoma brain metastases, nomogram, prognosis

## Abstract

**Background:**

Estimation of incidence and prognosis of melanomas with brain metastases (MBM) at initial diagnosis based on a large cohort is lacking in current research. This study aims to construct an effective prognostic nomogram for newly diagnosed MBM.

**Materials and Methods:**

Patients diagnosed with melanomas from Surveillance, Epidemiology, and End Results program between 2010 and 2014 were enrolled in our study. Risk factors predicting brain metastases (BM) were identified using logistic regression analysis. Cox regression analysis was performed to identify prognostic factors of overall survival (OS). Nomogram for estimating 6‐, 9‐, and 12‐month OS was established based on Cox regression analysis. The discriminative ability and calibration of the nomogram were tested using C statistics, calibration plots, and Kaplan‐Meier curves.

**Results:**

Sixty‐two thousand three hundred and sixty‐nine melanoma patients were enrolled, including 928 with BM. Sex, marital status, insurance status, subsite, surgery of primary sites, radiation, chemotherapy, bone metastases, liver metastases, and lung metastases were associated with MBM at initial diagnosis. On multivariable Cox regression, the following eight variables were incorporated in the prediction of OS: age, unmarried status, absence of surgery to primary sites or unknown, absence of radiation or unknown, absence of chemotherapy or unknown, with bone metastases, with liver metastases, and with lung metastases. The nomogram showed good predictive ability as indicated by discriminative ability and calibration, with the C statistics of 0.716 (95% CI, 0.695‐0.737).

**Conclusions:**

The incidence and prognosis of MBM patients were well estimated in this study based on a large cohort. The nomogram performed well and could be a useful tool to predict prognosis.

## Graphical abstract

This study provided population‐based estimates of the incidence and prognosis for patients of melanoma with brain metastases at initial diagnosis. The nomogram performed well and could be a useful tool to predict prognosis.

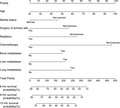

## 1 INTRODUCTION

In the United States, melanoma is the fifth most common cancer with a rapidly increasing incidence of 96 480 new cases in 2019.^1^ The 5‐year overall survival (OS) rate of cutaneous melanoma is 91.8% (2008‐2014).^2^ However, long‐term survival rate in patients with distant metastatic melanoma has been less than 10%,^2^ with a median survival time of only 6‐9 months.^3^ It is estimated that 40% of patients with melanoma initially present with localized disease, 9% with regional disease, and 4% with distant metastatic disease.^1^ Malignant cutaneous melanoma has the third highest incidence of brain metastasis among all types of cancer, following lung and breast cancers.^4^ According to previous studies, approximately 10% of patients with malignant melanoma and 40%‐60% of patients with metastatic melanoma ultimately develop brain metastases (BM).^5^, ^6^, ^7^ The related factors for melanoma with BM (MBM) incidence and prognosis such as age, depth of invasion, location, systematic disease, number of intracranial and extracranial metastases, etc, have all been reported in previous studies but remain controversial.^5^, ^8^, ^9^, ^10^ Nomogram survival prediction has been widely used in cancer research including hepatocellular carcinoma,^11^ adrenocortical carcinoma,^12^ and colorectal cancer.^13^ Yet, to our knowledge, it has not been reported as a related factor on MBM.

Therefore, our study aims to evaluate the incidence, risk, and prognostic factors of newly diagnosed MBM patients using the Surveillance, Epidemiology, and End Results (SEER) database. Nomogram survival prediction was visualized using Cox regression model due to its ease of use and ability to facilitate management‐related decision‐making.^14^ To our knowledge, we have established the first nomogram to predict the probability of survival rate for MBM.

## 2 MATERIALS AND METHODS

### 2.1 Patient

**Figure 1:**
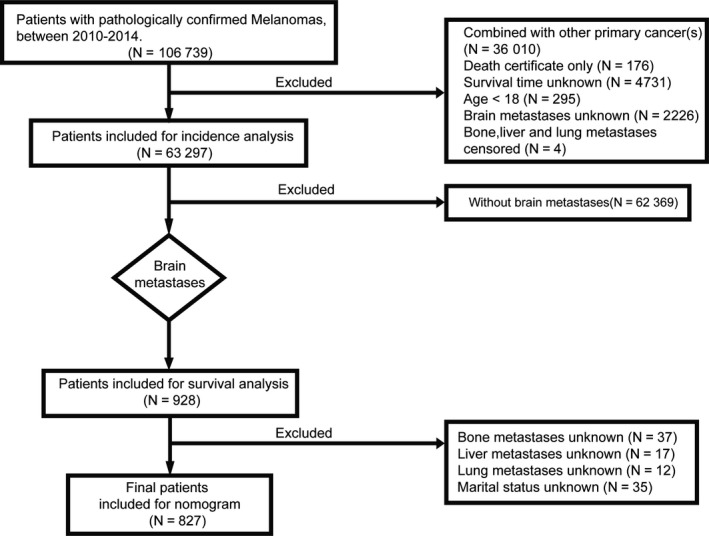
Flowchart of patient selection

Information about BM of melanoma patients at initial diagnosis from the SEER program between 2010 and 2014 was obtained. From the SEER database, 106 739 patients pathologically diagnosed with melanomas were identified. The followings were the exclusion criteria: (a) patients with other primary cancer(s) (N = 36 010); (b) patients diagnosed at autopsy (N = 176); (c) patients with missing or incomplete information about survival time (N = 4731); BM (N = 2226); bone, liver, and lung metastases (N = 4); (d) patients younger than 18 years old (N = 295). Based on these criteria, 63 297 patients remained for incidence analysis. But only 928 patients were diagnosed with MBM and used for survival analysis. Patients with unknown factors (N = 101) were removed; and therefore, only 827 patients were eligible for nomogram prediction (Figure 1). Informed consent was not required because SEER data contained no personal identifying information. The variables included in the study were age, sex, race, marital status, insurance status, subsite, surgery of primary sites, radiation, chemotherapy, bone metastases, liver metastases, lung metastases, BM, and survival months. Eight hundred and twenty‐seven MBM patients were then randomly divided into two groups: the training set (N = 414) and the validation set (N = 413). Training cohort was used to construct the nomogram and validation cohort was served as validation. There was no significant difference between the two groups (Table 1).

**Table 1:** Characteristics of patients between the training cohort and the validation cohort

	Training cohort (N/%)	Validation cohort (N/%)	*P*‐value
Age, mean ± SD (IQRs), y	60 ± 14 (52‐70)	60 ± 14 (52‐72)	.410
Marital status			.504
Married	223 (26.965)	233 (28.174)	
Not married	191 (23.096)	180 (21.765)	
Surgery of primary site(s)			.421
No/unknown	336 (40.629)	345 (41.717)	
Yes	78 (9.432)	68 (8.222)	
Radiation			.192
None/unknown	122 (14.752)	104 (12.576)	
Yes	292 (35.308)	309 (37.364)	
Chemotherapy			.542
No/unknown	275 (33.253)	265 (32.044)	
Yes	139 (16.808)	148 (17.896)	
Bone metastases			.811
No	326 (39.42)	329 (39.782)	
Yes	88 (10.641)	84 (10.157)	
Liver metastases			.375
No	312 (37.727)	323 (39.057)	
Yes	102 (12.334)	90 (10.883)	
Lung metastases			.548
No	185 (22.37)	175 (21.161)	
Yes	229 (27.69)	238 (28.779)	

Abbreviations: IQRs, interquartile ranges; SD, standard deviation.

### 2.2 Statistical analysis

Whole numbers and proportions were used to describe categorical variables, and means with interquartile ranges (IQRs) were used to describe continuous variables unless indicated otherwise. Chi‐square test and Fisher's exact test for categorical variables, and Student's *t* test for continuous variables were applied to compare baseline characteristics. Multivariable logistic regression was calculated to determine the potential risk factors associated with BM. Overall survival was defined as the length of time from diagnosis to any cause of death. Kaplan‐Meier method was used to calculate the OS, and differences were examined by the log‐rank test. Variables associated with OS were collected based on clinical importance and predictors identified in previous articles. Cox regression analysis was performed to identify the associations of relevant clinicopathological variables with OS. Hazard ratios (HR) with 95% confidence intervals (CIs) were then calculated.^15^ The validated variables were incorporated into the nomogram to predict the probability of 6‐, 9‐, and 12‐month OS rates for patients with melanomas and BM at initial diagnosis using the rms package in R software.^16^ The performance of the model was assessed by the discriminating ability and calibration ability. The discrimination of this model was assessed by C statistics.^17^ Calibration was evaluated using a calibration plot, which compared the actual probabilities and the nomogram‐predicted probabilities. Bootstrap sample^12^ was used to correct for overfitting bias. Kaplan‐Meier curves were used to further assess calibration by plotting over stratified patient scores predicted from nomograms in the dataset.^12^ A two‐tailed *P* < .05 was considered statistically significant. Statistical analyses were performed with the R 3.4.3 software.

## 3 RESULTS

### 3.1 Incidence

**Figure 2:**
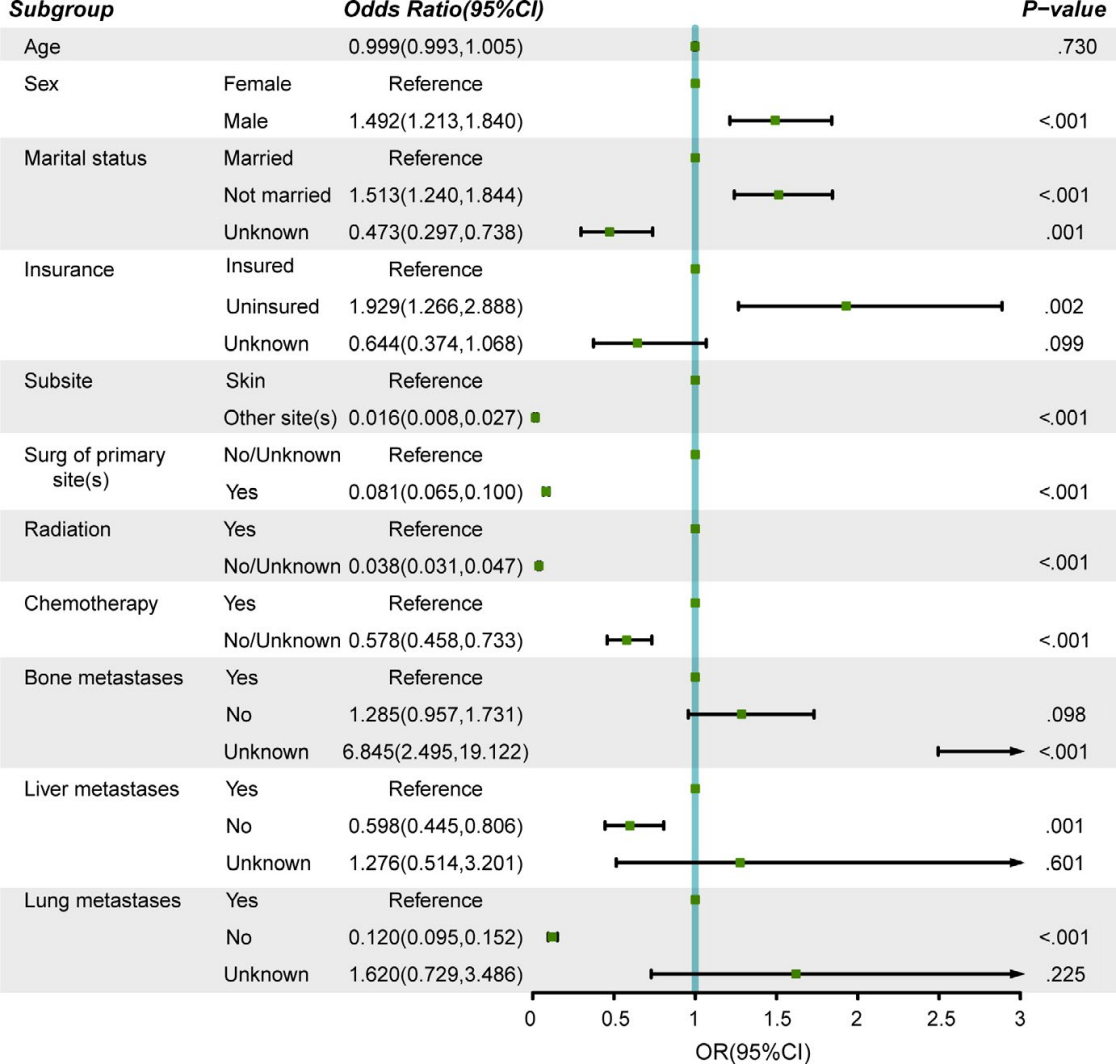
Multivariate logistic regression for melanoma with brain metastases. CI, confidence interval; Surg, surgery

The baseline characteristics of newly diagnosed melanoma patients with (or without) BM are shown in Table 2. Nine hundred and twenty‐eight patients presented with BM, accounting for 1.47% of the whole study cohort. The number of melanoma patients increased from 2010 to 2014 (*P* = .019). The mean age for MBM patients was 61 years (IQRs, 52‐71 years). Of these 928 patients, 73.6% were male and 97.7% were of white race. Approximately 90% of the patients had insurance. As indicated, patients were more likely to be married, to have higher rates of primary tumor surgery, lower rates of bone, liver and lung metastases; these patients also were more likely to have lower rates of undergoing radiation and chemotherapy (*P* < .001). Additionally, skin was the most common primary site of melanomas (97.5%). On multivariable logistic regression (Figure 2), male (vs female; odds ratio [OR] 1.492; 95% CI 1.213‐1.840; *P* < .001), unmarried status (vs married; OR 1.513; 95% CI 1.240‐1.844; *P* < .001), uninsured status (vs insured; OR 1.929; 95% CI 1.266‐2.888; *P* = .002), and unknown bone metastases (vs bone metastases; OR 5.915; 95% CI 2.119‐16.928; *P* < .001) were related to greater odds of the presence of BM at diagnosis. Unknown marital status (vs married; OR 0.473; 95% CI 0.297‐0.738; *P* = .001), other sites (vs skin melanomas; OR 0.016; 95% CI 0.008‐0.027; *P* < .001), surgery to primary sites (vs no/unknown; OR 0.081; 95% CI 0.065‐0.100; *P* < .001), absence of radiation or unknown (vs radiation; OR 0.038; 95% CI 0.031‐0.047; *P* < .001), absence of chemotherapy or unknown (vs chemotherapy; OR 0.578; 95% CI 0.458‐0.733; *P* < .001), without liver metastases (vs liver metastases; OR 0.598; 95% CI 0.445‐0.806; *P* = .001) and without lung metastases (vs lung metastases; OR 0.120; 95% CI 0.095‐0.152; *P* < .001) were connected with lower odds of having BM. According to the multivariable model, age was not associated with BM at diagnosis. Significant results are presented in Figure 2.

**Table 2:** Patient characteristics of melanomas

	Brain metastases		*P*‐value	MST (95% CI)
	No (%)	Yes (%)		
Year of diagnosis			**	
2010	11 557 (18.53)	180 (19.40)		4 (4, 5)
2011	11 635 (18.66)	188 (20.26)		3 (3, 5)
2012	12 487 (20.02)	148 (15.95)		4 (3, 6)
2013	12 837 (20.58)	184 (19.83)		6 (4, 6)
2014	13 853 (22.21)	228 (24.57)		5 (4, 7)
Age, mean ± SD (IQRs), y	59 ± 16 (49‐70)	61 ± 14 (52‐71)	***	—
Sex			***	
Female	28 033 (44.947)	245 (26.401)		4 (3, 5)
Male	34 336 (55.053)	683 (73.599)		4 (4, 5)
Race			***	
White	57 691 (92.50)	907 (97.74)		4 (4, 5)
Black	331 (0.53)	3 (0.32)		5 (2, NR)
Other	599 (0.96)	17 (1.83)		6 (4, NR)
Unknown	3748 (6.01)	1 (0.11)		NA
Marital status			***	
Married	31 326 (50.23)	493 (53.13)		5 (4, 6)
Not married	15 053 (24.14)	397 (42.78)		4 (3, 5)
Unknown	15 990 (25.64)	38 (4.10)		4 (1, 7)
Insurance			***	
Insured	48 386 (77.58)	843 (90.84)		4 (4, 5)
Uninsured	1275 (2.04)	60 (6.47)		3 (2,6)
Unknown	12 708 (20.38)	25 (2.69)		2 (2, 7)
Subsite			***	
Skin	59 909 (96.06)	915 (98.60)		4 (4, 5)
Other sites	2460 (3.94)	13 (1.40)		5 (2, NR)
Surgery of primary site(s)			***	
Yes	57 854 (92.76)	164 (17.67)		7 (6, 9)
No/unknown	4515 (7.24)	764 (82.33)		4 (3,4)
Radiation			***	
Yes	2455 (3.94)	670 (72.20)		5 (4, 6)
No/unknown	59 914 (96.06)	258 (27.80)		2 (2, 3)
Chemotherapy			***	
Yes	1155 (1.85)	319 (34.38)		7 (6, 8)
No/unknown	61 214 (98.15)	609 (65.63)		3 (3, 3)
Bone metastases			***	
Yes	549 (0.88)	190 (20.47)		5 (4, 6)
No	62 696 (99.05)	701 (75.54)		2 (1, 4)
Unknown	52 (0.083)	37 (3.99)		3 (3, 4）
Liver metastases			***	
Yes	460 (0.74)	213 (22.95)		5 (5, 6)
No	61 887 (99.23)	675 (72.74)		2 (2, 4)
Unknown	22 (0.04)	40 (4.31)		3 (2, 3)
Lung metastases			***	
Yes	704 (1.13)	513 (55.28)		6 (5, 8)
No	61 633 (98.82)	380 (40.95)		3 (2, 6)
Unknown	32 (0.05)	35 (3.77)		4 (3, 4)

Abbreviations: CI, confidence interval; MST, median survival time; NA, not applicable; NR, not reached.

** *P* < .05.

*** *P* < .001.

### 3.2 Survival analysis

**Figure 3:**
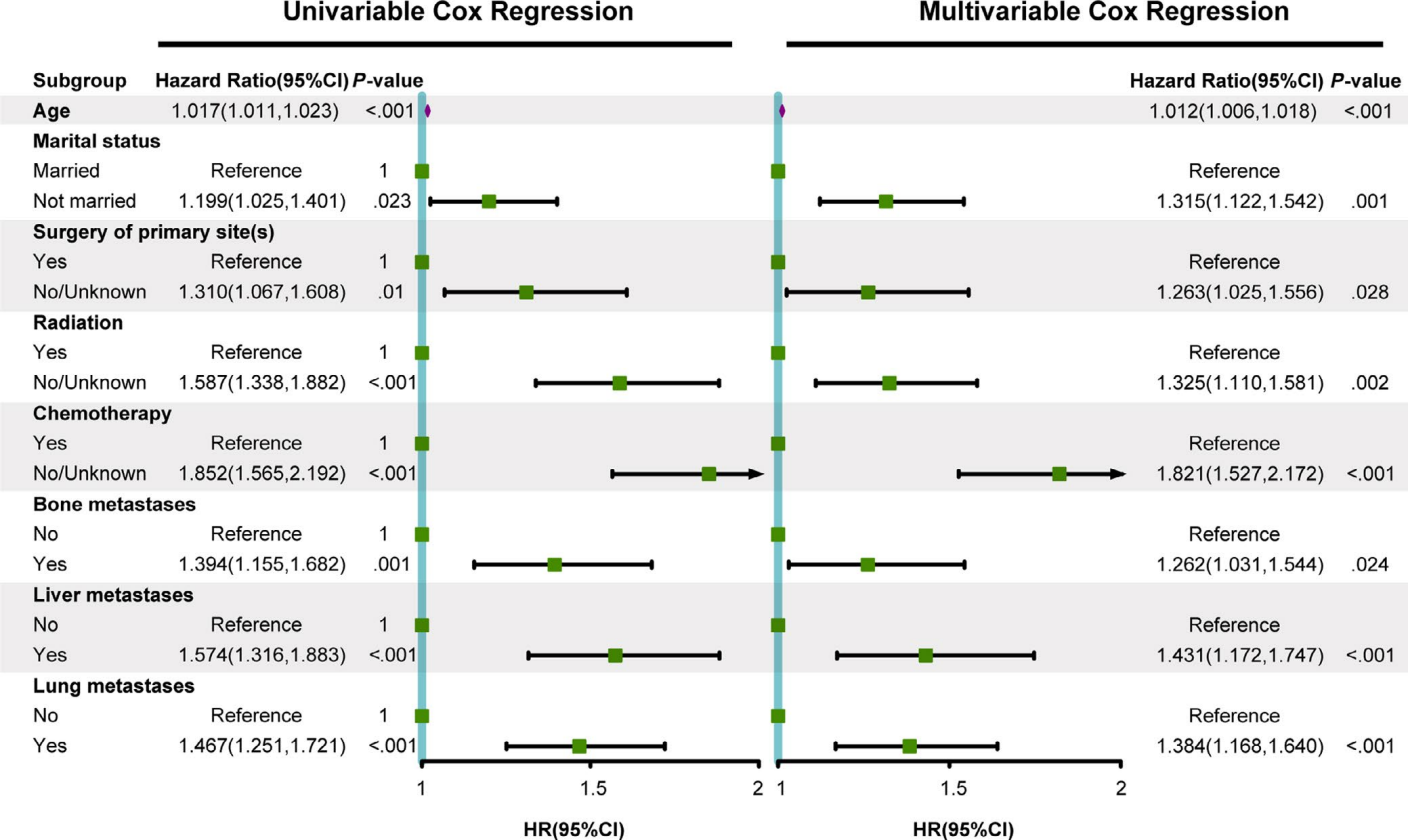
Univariable and multivariate Cox regression for analyzing prognostic factors for patients with melanoma and brain metastases at initial diagnosis

The median follow‐up time was 23 months. Median survival time for the BM study group was 4 months. The 6‐, 9‐ and 12‐month OS percentages were 38.3% (95% CI 35.2%‐41.7%), 29.0% (95% CI 26.0%‐32.3%), and 21.6% (95% CI 18.9%‐24.8%), respectively. The following variables in the Cox regression, analyzed by backward stepwise selection using the Akaike information criterion, were associated with OS: age, marital status, surgery of primary sites, radiation, chemotherapy, bone metastases, liver metastases, and lung metastases (Figure 3). On multivariable analysis, age (HR 1.012; 95% CI 1.006‐1.018; *P* < .001), unmarried status (HR 1.315; 95% CI 1.122‐1.542; *P* = .001), absence of surgery to primary sites or unknown (HR 1.263; 95% CI 1.325‐1.556; *P* = .028), absence of radiation or unknown (HR 1.325; 95% CI 1.110‐1.581; *P* = .002), absence of chemotherapy or unknown (HR 1.821; 95% CI 1.527‐2.172; *P* < .001), with bone metastases (HR 1.262; 95% CI 1.031‐1.544; *P* = .024), with liver metastases (HR 1.431; 95% CI 1.172‐1.747; *P* < .001), and with lung metastases (HR 1.384; 95% CI 1.168‐1.640; *P* < .001) were each independently associated with OS (Figure 3).

### 3.3 Nomogram and model performance

**Figure 4:**
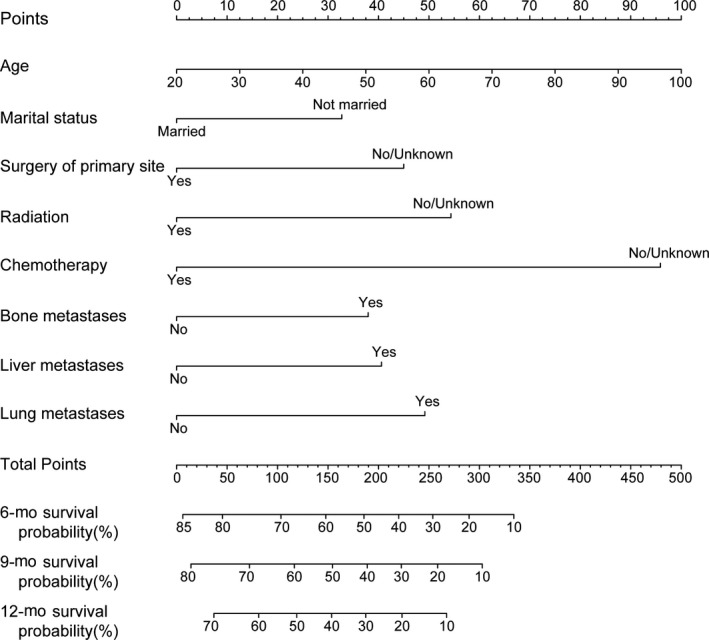
Nomogram predicting survival in patients with melanoma and brain metastases at initial diagnosis. The nomogram to predict overall survival was created based on eight independent prognostic factors

**Figure 5:**
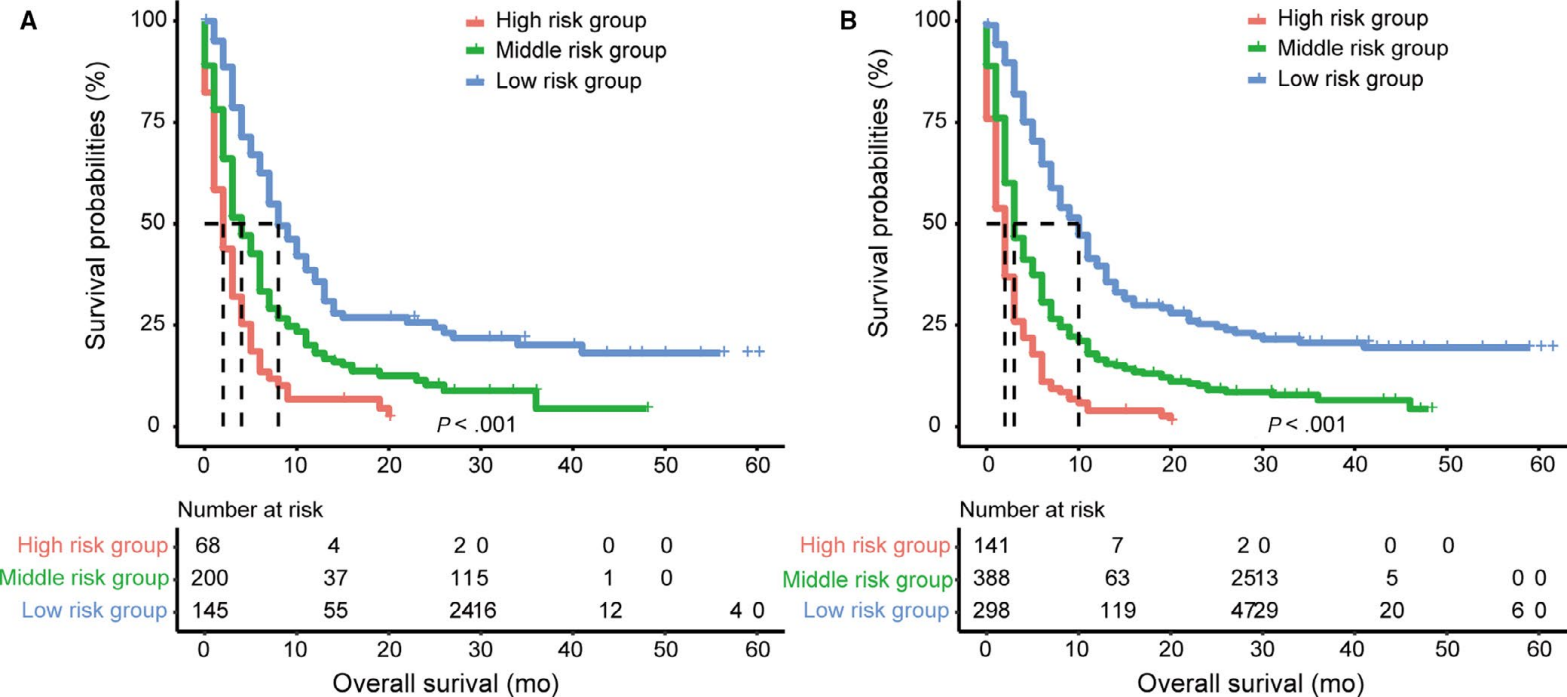
Kaplan‐Meier curves demonstrating survival in patients with melanoma and brain metastases at initial diagnosis according to groups of predicted survival. A, Kaplan‐Meier curves demonstrating survival of the validation cohort; B, Kaplan‐Meier curves demonstrating survival of the whole cohort

**Figure 6:**
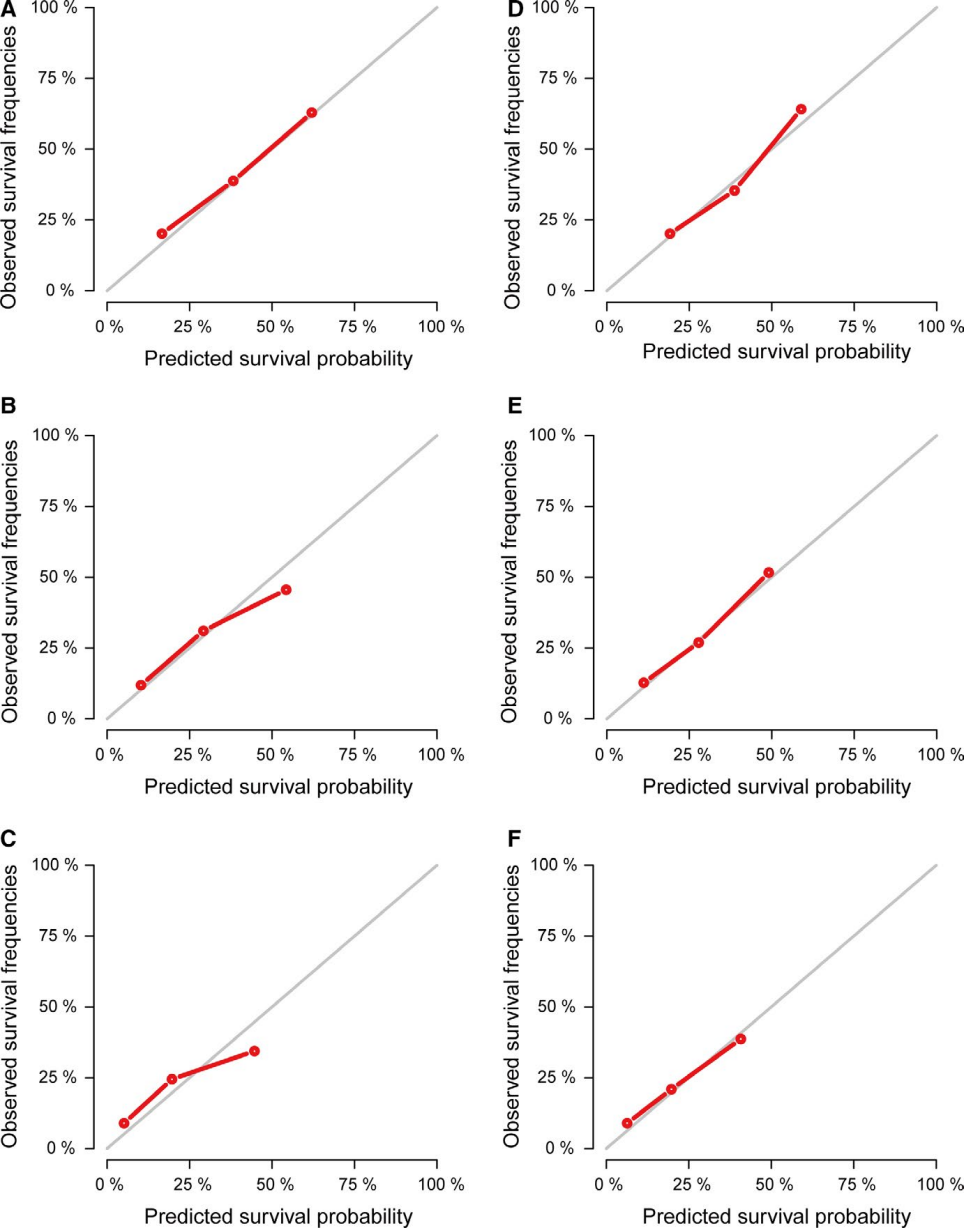
Calibration plot comparing predicted and actual survival probabilities at 6‐, 9‐, and 12‐mo follow‐up. A‐C, The plot for the prediction of 6‐, 9‐, and 12‐mo overall survival between training cohort and validation cohort. D‐F, The 1000‐sample bootstrapped calibration plot for the prediction of 6‐, 9‐, and 12‐mo overall survival by validation. The red line represents the ideal fit; the black line represents the actual fit

Nomogram predicting OS of the melanoma patients with BM is presented in Figure 4. The following eight independent prognostic factors were incorporated into the nomogram: age, marital status (married or unmarried), surgery to primary sites (yes or no/unknown), radiation (yes or no/unknown), chemotherapy (yes or no/unknown), bone metastases (yes or no), liver metastases (yes or no), and lung metastases (yes or no). Lower total points based on the sum of the nomogram were related to a better prognosis. For example, a melanoma patient with 60 years of age, unmarried status, evidence of bone metastases, and surgery of primary sites would have a total of 120 points assigned (50 points for age, 32.5 points for unmarried status, 0 points for surgery to primary sites, 37.5 points for bone metastases), for a predicted 6‐, 9‐, and 12‐month OS of 65.0%, 57.5%, and 50.0%, respectively. Kaplan‐Meier curves based on the predicted probability of OS were plotted to further evaluate the discriminative ability of the model. These curves were stratified by the predicted probability of the group calculated from the nomogram: low‐risk group, middle‐risk group, and high‐risk group. Patients of high‐risk group had a substantially worse outcome compared with patients of low‐risk group and middle‐risk group (*P* < .001) (Figure 5). The nomogram‐predicted median OS revealed good estimation when compared with the actual survival based on Kaplan‐Meier method. A C statistics of 0.716 (95% CI 0.695‐0.737) was used to assess the discrimination of the model. The accuracy of the model and potential model overfit were assessed by two approaches: (a) comparison between the training cohort, used to create the nomogram, with the validation cohort (Figure 6A‐C), (b) bootstrap validation with 1000 resamplings from the whole population (Figure 6D‐F). The calibration plots, displaying the probability of survival at 6, 9, and 12 months after diagnosis, showed a strong correlation between the nomogram‐predicted probabilities and the observed probabilities.

## 4 DISCUSSION

Lung cancer, breast cancer, and melanoma are the three most common sources of BM in patients.^18^ The lower morbidity of melanoma reflects the high brain metastasis tendency; and therefore, renders greater importance to developing more effective therapies. However, further understanding of the clinical characteristics, risk, and prognostic factors of BM in melanoma is still required to improve research in this area.

The salient and novel findings of our study were as follow. First, to our knowledge, this study contained the largest cohort of MBM patients. In the initial diagnosed melanoma cohort, 1.47% had BM. Melanoma patients who were presence with bone or lung metastases had greater opportunities of developing BM at diagnosis. Secondly, melanoma with BM was a fatal disease with a median OS of 4 months. Generally, with melanoma there appears to be a relationship between parenchymal CNS metastases and leptomeningeal metastasis (LM): 87%–96% of melanoma patients with BM are associated with LM.^19^, ^20^ Compared to the median OS of 4 months for MBM patients found in our study, outcomes for melanoma patients with LM are worse with a median OS of 6‐8 weeks without tumor‐specific treatment. On the other hand, LM‐directed treatment including targeted therapy and immunotherapy may only prolong the median OS to 1.7‐2.5 months.^19^, ^20^ However, further exploration of this comparison is limited due to the lack of detailed information about parenchymal CNS metastases and LM in the SEER database. Third, in this study, we constructed a nomogram that numerically facilitated individualized prediction of OS in melanoma patients with BM at initial diagnosis relied on patient‐related and tumor‐related factors. It can be used for patient‐consulting on prognostic information, as well as to help physician make individualized clinical decisions combining with the AJCC staging system. Indeed, when stratified into groups, the nomogram was able to identify distinct groups of patients having different risks of death. Most importantly, our nomogram presented good discriminative ability, with a C statistic of 0.716 (95% CI 0.695‐0.737). Accurate risk stratification of patients with melanoma is important due to the heterogeneity of patient prognoses.

This study is the first study to predict the prognosis of MBM through a nomogram. However, there are still several limitations to our study. First, the SEER database underestimates the total diagnosed cancer cases because it only collects information on newly diagnosed cancer cases. Consequently, patients who have BM later in their disease course would not be included in the data. This explains the lower proportion of BM, 1.47%, found in our study compared to that observed in the whole melanoma population, approximately 10%.^5^, ^21^, ^22^ It is important to know that the risk and prognostic factors of BM only reflect the characteristics of this cohort, which are not equal to those for the whole melanoma population. Therefore, further studies are needed to explore the potential differences between this cohort and those who were diagnosed with BM during follow‐up. The second study limitation is the unavailable information on the number of BM, Karnofsky performance status, comorbidities, extracranial disease, LM, aggregate brain tumor volume or BRAF status, some of which have been confirmed to be potential important prognostic indicators.^23^, ^24^, ^25^ Third, although our nomogram was validated using two methods and presented strong agreement between the nomogram‐predicted survival and the actual survival, external validation is needed in the future to validate the recommended nomogram.

## 5 CONCLUSION

In conclusion, our study developed a convenient nomogram, which may offer prognostic assessment for individual MBM. Additional studies are required to determine whether it can be applied to other patient groups.

## CONFLICT OF INTEREST

None declared.

## Data Availability

The data that support the findings of this study are openly available in the Surveillance, Epidemiology and End Results, https://seer.cancer.gov.
